# RNAs coordinate nuclear envelope assembly and DNA replication through ELYS recruitment to chromatin

**DOI:** 10.1038/s41467-017-02180-1

**Published:** 2017-12-14

**Authors:** Antoine Aze, Michalis Fragkos, Stéphane Bocquet, Julien Cau, Marcel Méchali

**Affiliations:** 1 Institute of Human Genetics, UMR 9002, CNRS and the University of Montpellier, Replication and Genome Dynamics, 141 rue de la Cardonille, 34396 Montpellier, France; 2 Institute of Human Genetics, UMR 9002, CNRS and the University of Montpellier, Montpellier RIO Imaging, 141 rue de la Cardonille, 34396 Montpellier, France; 3 Present Address: Institut Gustave Roussy, Genetic Stability and Oncogenesis Department, 39 rue Camille Desmoulins, 94805 Villejuif, France

## Abstract

Upon fertilisation, the sperm pronucleus acquires the competence to replicate the genome through a cascade of events that link chromatin remodelling to nuclear envelope formation. The factors involved have been partially identified and are poorly characterised. Here, using *Xenopus laevis* egg extracts we show that RNAs are required for proper nuclear envelope assembly following sperm DNA decondensation. Although chromatin remodelling and pre-replication complex formation occur normally, RNA-depleted extracts show a defect in pre-RC activation. The nuclear processes affected by RNA-depletion included ELYS recruitment, which accounts for the deficiency in nuclear pore complex assembly. This results in failure in chromatin relaxation as well as in the import and proper nuclear concentration of the S-phase kinases necessary for DNA replication activation. Our results highlight a translation-independent RNA function necessary for the parental genome progression towards the early embryonic cell cycle programme.

## Abstract (web summary)

The factors that link chromatin remodelling to nuclear envelope formation in the sperm pronucleus are not fully characterised. Here, the authors show that in RNA-depleted *Xenopus laevis* egg extracts, ELYS recruitment and nuclear pore complex formation are impaired, resulting in defective nuclear processes.

## Introduction

In eukaryotic cells, the nuclear envelope (NE) isolates the genome from the cytoplasmic compartment by forming a barrier around the nucleus, where gene transcription and DNA replication take place in a coordinated manner during the cell cycle interphase^1^. During mitosis, this physical barrier is disrupted and the genome is completely reorganised to allow chromosome segregation^2^. After mitosis, a new NE is formed around chromosomes^3^. This requires the assembly of nuclear pore complexes (NPCs) that allow the exchange of molecules between nucleus and cytoplasm, leading to nuclear expansion. This process also contributes to the dynamic organisation of the chromatin structure for transcription and DNA replication^4–7^.

NE assembly in *Xenopus laevis* egg extracts mimics events occurring after fertilisation, when nucleoplasmin-dependent decondensation of sperm chromatin occurs rapidly and results in the replacement of sperm protamines by maternal histones stored in the egg^8^. NPC assembly is initiated by the nucleoporin (NUP) ELYS (MEL-28/AHCTF1)^9–11^. Then, the NE is reconstituted and the nuclear volume increases, owing to the import of nuclear proteins, including those involved in the activation of DNA replication^10,12,13^. Concomitantly with the acquisition of the nuclear structure, replication origins become licensed for DNA replication after the loading onto chromatin of the pre-replication complex (pre-RC) factors, including the replicative helicase MCM2-7. Successful pre-RC assembly seems to require sperm decondensation, because nucleoplasmin is needed for binding of ORC, the earliest known pre-RC component recruited to chromatin^14^. Then, the transition from pre-RC to pre-initiation complex (pre-IC) is induced by S-phase kinases, and DNA replication is activated only after the complete assembly of a functional nucleus and the local concentration of replication nuclear factors^10,12,13^. Therefore, nuclear membrane formation and DNA replication are specifically interconnected.

Recently, it has been reported that DNA packaging in nucleosomes is essential for NPC assembly and for the correct recruitment of ELYS to chromatin in *X*. *laevis* egg extracts and also in mouse zygotes^15,16^. ELYS might also be involved in chromatin decondensation that is linked to DNA replication in *Caenorhabditis elegans* early embryos^17^. As ELYS interacts with lamins and MCM2−7^9-11^, it could be one of the main factors associated with the NE to coordinate chromatin compaction up to chromosome decondensation and assembly of the proteins involved in S-phase activation. However, it is not known what aspect of chromatin organisation requires ELYS during NE assembly.

Non-coding RNAs are involved in the regulation of chromatin dynamics as structural or regulatory RNAs^18,19^. For instance, a subset of chromatin-associated RNAs maintains the higher-order structures of euchromatic and heterochromatic regions in vertebrates^20^. Moreover, various non-coding RNAs, such as Y-RNAs, might regulate functions required for DNA replication in metazoans^21,22^. Nonetheless, the generation of these RNAs is transcription-dependent and therefore, investigating their functions by disturbing their synthesis could also affect gene expression programmes. This problem can be circumvented by using *X*. *laevis* egg extracts. This in vitro system is transcription-independent^23^ and reproduces most of the events linked to chromatin reorganisation and DNA replication activation during early development. This is possible because *X*. *laevis* eggs contain large amounts of the components necessary for these processes because they are stored during oogenesis. These components allow early development to proceed in the absence of transcription for twelve cell cycles. Here, we used this system to investigate the involvement of RNAs in chromatin reorganisation and DNA replication activation. We found that when sperm nuclei were incubated in RNA-depleted *X*. *laevis* egg extracts, DNA replication was strongly inhibited. This defect was not owing to alterations in pre-RC formation, but was linked to a defect in the activation of DNA replication origins. Moreover, nuclear organisation was also disrupted, and lamin LIII could not accumulate in the nucleus. NPCs failed to assemble at the nuclear membrane, and proteins could not be imported in the nucleus. By investigating the molecular mechanisms responsible for this replication defect, we found that RNAs are required to favour ELYS accessibility to chromatin and for NPC assembly, two precursor steps before DNA replication.

## Results

### RNA depletion inhibits replication but not Pre-RC formation

To investigate whether RNAs are involved in the processes leading to pronucleus formation and activation of DNA replication following fertilisation in metazoans, we used the in vitro system derived from *X. laevis* low-speed egg extracts (LSE), where nuclear formation and semi-conservative DNA replication of the added demembranated sperm nuclei occur in the absence of both transcription and translation^23,24^. Moreover, all the experiments described here were performed in the presence of cycloheximide, a translation inhibitor.

**Fig. 1:**
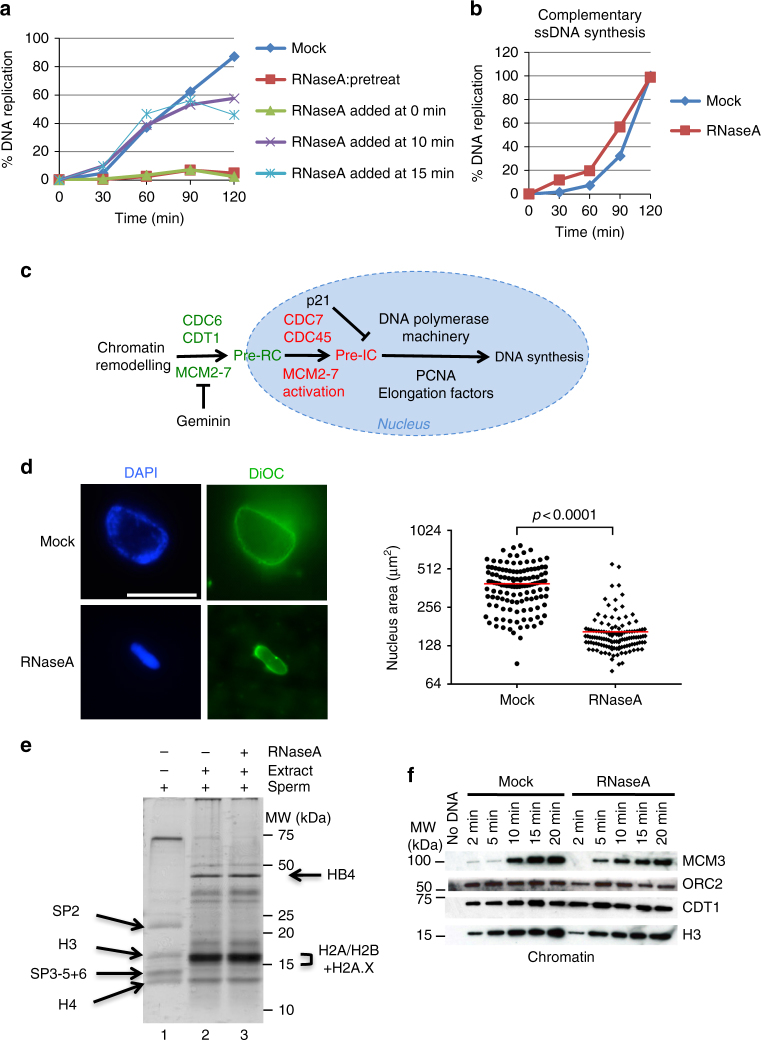
Incubation with RNase A inhibits DNA replication but not pre-RC assembly. **a** Replication kinetics of LSEs incubated with RNase A before (pretreat), at the same time (0 min), and 10 or 15 min after addition of sperm nuclei. **b** M13 ssDNA replication is not affected by LSE pre-incubation with RNase A, **c** Scheme illustrating the steps leading to the assembly of the complexes involved in the licensing reaction (green) and in DNA replication activation (red). The blue circle highlights reactions occurring in the nucleus. **d** Sperm nuclei incubated in mock or RNase A-treated LSE for 60 min were fixed in the presence of DiOC_6_ (green) and DAPI (blue). Scale bar, 17 μm. The nuclear size of 125 nuclei per condition was measured based on the DAPI staining area (right panel). Red bar, mean value. A two-tailed Student’s *t*-test was performed to determine the *p*-values. **e** Histones from sperm nuclei were isolated by acid extraction before and 5 min after incubation in mock or RNase A-treated extracts. DNA-bound proteins were then analysed by gel electrophoresis and silver staining. **f** Time-course analysis of pre-RC assembly in HSE (chromatin purification followed by western blotting with specific antibodies)

We first asked whether LSE pre-incubation with RNase A before addition of sperm nuclei affected sperm DNA replication. RNase A strongly inhibited DNA synthesis (Fig. 1a and Supplementary Fig. 1a) in a concentration-dependent manner (Supplementary Fig. 1b, c), while Flag peptides (used as control) or heat-inactivated RNase A did not affect DNA replication kinetics (Supplementary Fig. 1d, e). DNA replication was also not affected if sperm nuclei were first RNase A-treated before transfer in an RNasin-containing LSE (to block RNase activity) (Supplementary Fig. 1f). This indicates that the destruction of RNAs present in sperm nuclei did not explain LSE RNase sensitivity. Finally, recombinant H12K/H119Q RNase A, a mutant that is enzymatically inactive, did not inhibit DNA replication, differently from recombinant wild-type RNase A that was purified in parallel (Supplementary Fig. 2a-c).

Checkpoint activation was unlikely to be involved in DNA replication inhibition by RNase A because CHK1 was not phosphorylated in extracts pre-incubated with RNase A, differently from those incubated with aphidicolin, an inhibitor of DNA synthesis (Supplementary Fig. 3a). Moreover, the checkpoint inhibitor caffeine did not reduce the DNA replication defect (Supplementary Fig. 3b). Interestingly, DNA synthesis was not affected when RNase A was added 10–15 min after addition of sperm nuclei into the extract, when DNA synthesis was not yet initiated (Fig. 1a). This result suggests that RNAs are dispensable during DNA synthesis, but are required at an earlier stage of DNA replication.

To determine whether RNAs were required for complementary DNA strand synthesis, we used M13 ssDNA, because its complementary strand can be efficiently synthesised and assembled into chromatin in *X*. *laevis* egg extracts, in a reaction that mimics events at the replication fork^25^. RNase A did not affect M13 DNA synthesis (Fig. 1b), indicating that endogenous RNAs are not required for priming or elongation of DNA strands.

To identify the DNA replication step affected by RNA depletion, we investigated the events that occur after fertilisation and that precede or are concomitant with DNA synthesis initiation (Fig. 1c). First, we assessed the ability of sperm DNA to assemble nuclei in the absence of RNAs (Fig. 1d). Nuclei formed in RNase A-treated egg extracts became surrounded by well-fused membranes, similarly to nuclei in mock-treated extracts, as shown by DiOC_6_ staining. However, these nuclei were remarkably smaller than control nuclei (Fig. 1d) or nuclei assembled in extracts pre-incubated with the catalytically inactive RNase A mutant (Supplementary Fig. 4a). This could be explained by a defect during sperm chromatin decondensation. Therefore, we evaluated the remodelling efficiency of chromatin from sperm nuclei incubated in RNase A-treated extracts (Fig. 1e). To detect the protamines and histones that compose the chromatin of sperm nuclei, we isolated acid-soluble chromatin-associated proteins, which include also histones, and separated them by SDS-PAGE (see Methods) (Fig. 1e, lane 1). Incubation of sperm nuclei in mock-treated egg extracts led to protamine removal (lane 2) and chromatin remodelling by incorporation of the maternal histones H2A and H2B, as expected^26^. We obtained similar results when sperm nuclei were incubated in RNase A-treated egg extracts (lane 3), suggesting that protamine elimination and maternal histone incorporation occurred normally. This result excluded a possible effect of polyanion imbalance in nucleosome assembly in the extract (see also later).

In all eukaryotic cells, initiation of DNA replication occurs in two successive steps: pre-RC assembly at origins in G1, and pre-RC activation in S-phase (Fig. 1c and^27^ for a review). To assess whether pre-RCs assembled normally in the absence of RNAs, we took advantage of high-speed egg extracts (HSE)^25^ that fully recapitulate pre-RC formation, but in which DNA synthesis is not activated owing to the absence of the nuclear membrane fraction^28^. We isolated chromatin after addition of sperm DNA to HSE, and analysed the bound pre-RC components by western blotting (Fig. 1f). Incubation in RNase A-treated HSE did not affect ORC2, CDT1 or MCM3 assembly onto chromatin, indicating that RNAs are not required for pre-RC formation.

From these results, we conclude that RNA depletion inhibits DNA synthesis, but does not affect remodelling of paternal histones by maternal histones and pre-RC formation.

### RNAs are required for lamin LIII assembly on chromatin

**Fig. 2:**
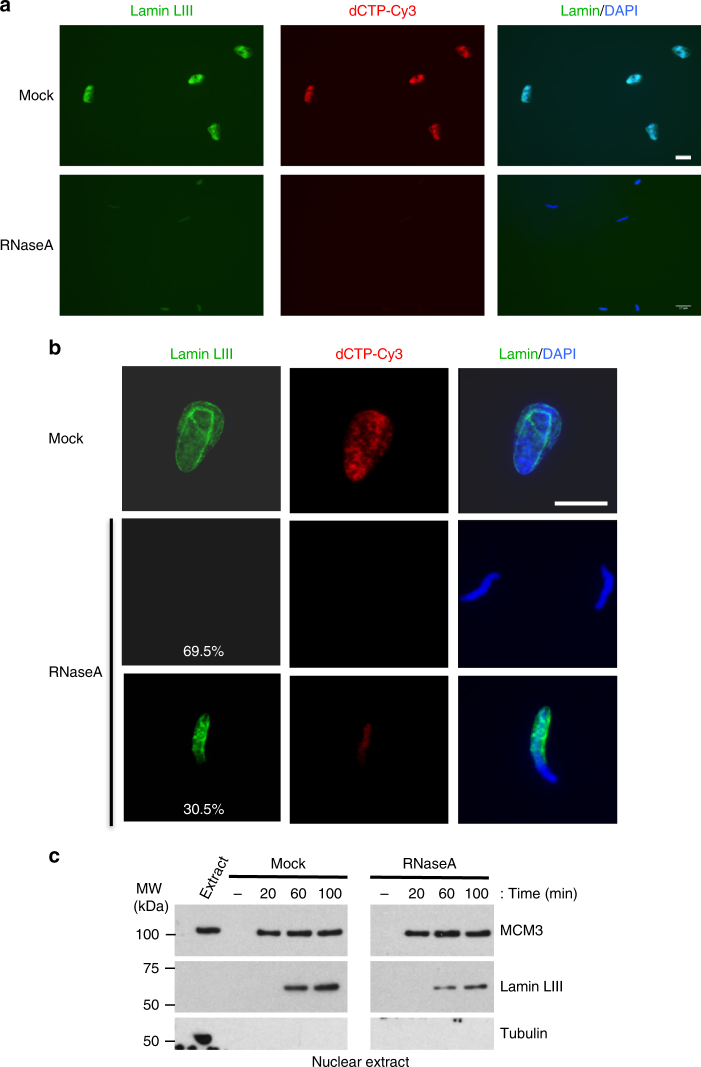
The nuclear architecture is affected when assembled in RNA-depleted egg extracts. **a** Immunofluorescence analysis of lamin LIII accumulation (green) in a representative population of nuclei fixed after incubation in mock- or RNase A-treated *X*. *laevis* egg extracts for 60 min. DNA synthesis was monitored by dCTP-Cy3 incorporation (red), and DNA was stained with DAPI (blue). Scale bar, 17 μm. **b** Higher magnification of representative nuclei from the experiments described in **a**. Lamin LIII could not be detected in 70% of nuclei assembled in RNA-depleted egg extracts and its accumulation was abnormal in the other 30%. Scale bar, 17 μm. **c** Western blot analysis of nuclear extracts prepared from sperm nuclei at different time points during incubation in mock-depleted or RNA-depleted extracts

In vivo, DNA replication is intrinsically linked to the structural organisation of the nucleus, including NE, chromatin and replication foci (^27^ for a review). The small size of nuclei assembled in RNase A-treated extracts suggested a global change in chromatin structure. The nuclear lamina, a network of intermediate filaments that anchors NPCs to the NE, is one of the main components involved in chromatin organisation. Thus, we examined the nuclear accumulation of lamin LIII (also known as lamin B3), the major lamin in *X*. *laevis* eggs. Immunofluorescence analyses (Fig. 2a) showed that lamin LIII accumulation was strongly reduced in most of the nuclei formed in RNase A-treated egg extracts. Only 30% of nuclei from RNase A-treated extracts assembled a nuclear lamina that, however, was abnormal and incomplete (Fig. 2b). Western blot analysis confirmed the defect in lamin LIII assembly in RNA-depleted extracts (Fig. 2c).

### RNA depletion targets S-phase kinase-dependent replication

**Fig. 3:**
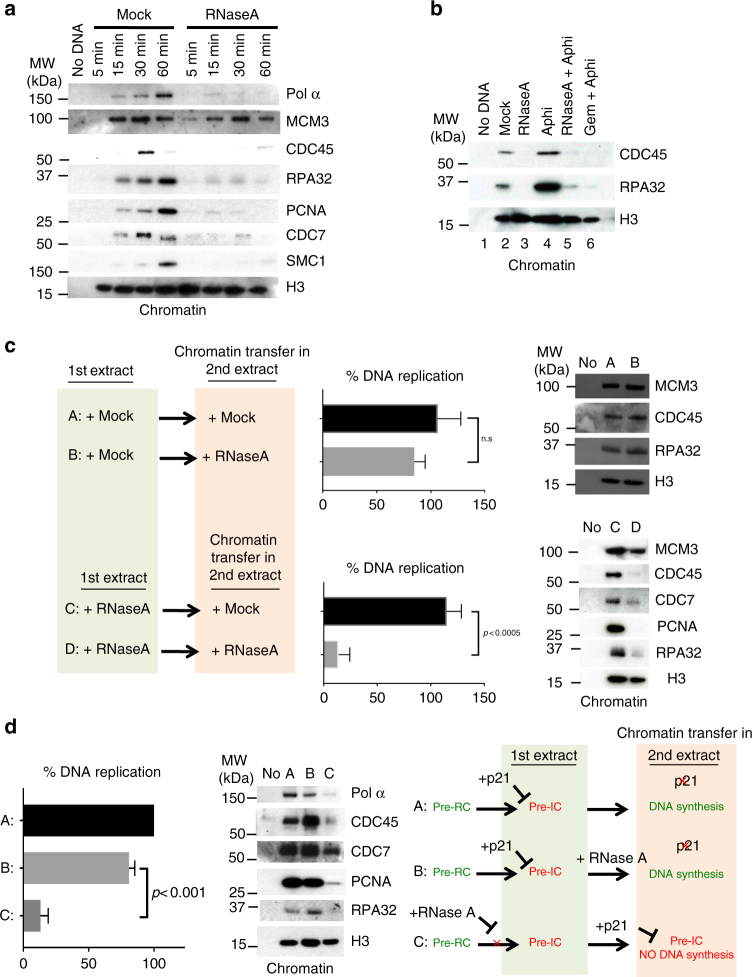
RNA is required for pre-IC assembly but is dispensable after pre-IC formation. **a** Time-course analysis of the recruitment of pre-IC and replication fork components to sperm chromatin in mock- and RNase A-treated LSEs. Histone H3 was used as a loading control. **b** Western blot showing the absence of DNA unwinding in RNase A-treated extracts. Sperm chromatin was purified and analysed 30 min after incubation in LSE. Incubation with 100 ng/μl aphidicolin (Aphi) resulted in RPA overloading following DNA polymerase inhibition and uncoupling from the MCM2-7 helicase activity. Geminin was used as negative control. **c** Chromatin transfer experiments showing that DNA replication is inhibited by RNA depletion before pre-IC formation in a reversible manner. Lanes A and B, chromatin was assembled in a mock-treated LSE for 40 min to allow pre-IC formation, and then transferred to an RNase A-treated extract or another mock extract (control). The graph shows the percentage of DNA replication at 120 min after chromatin transfer (*n* = 5), and the right panel shows the loading of replication proteins onto chromatin. Lanes C and D, the DNA replication defect is abolished when nuclei assembled in RNase A-treated extracts are transferred into mock-treated extracts C, but not after transfer into a new RNAse A-treated extract D. Error bar represents the standard deviation (SD). *P*-values were determined with the two-tailed Student’s *t*-test. n.s.: not significant. **d** RNA depletion blocks S-phase-dependent kinase activation. Lanes A and B, sperm nuclei were initially incubated in p21-treated LSE (for 30 min) and then transferred to mock- or RNase A-treated LSE, respectively. C: Chromatin assembled in an RNase A-treated LSE extract was transferred in a p21-treated extract. DNA replication levels (*n* = 3) and binding of replication factors to chromatin were monitored as described in **a**. Error bar shows the SD. A two-tailed Student’s *t*-test was performed to calculate the *p*-values

In vivo, nuclear lamina assembly around sperm chromatin is required to support the recruitment of replication factors to chromatin^29,30^. We, therefore, monitored the loading of replication factors on sperm DNA after pre-RC assembly. Pre-IC assembly on chromatin and replication origin activation are coordinated through the kinase activities of CDC7/DBF4 and cyclin-dependent kinases (CDKs)^31–35^ (Fig. 1c). Moreover, in *X*. *laevis* the pre-IC component CDC45 requires both CDC7 and CDKs for its recruitment to chromatin^36^. The binding to chromatin of all factors required after pre-RC formation was impaired in RNase A-treated egg extracts compared with mock-treated samples (Fig. 3a). CDC7 and CDC45 were not loaded on chromatin and the subsequent recruitment of elongation factors, such as PCNA, did not occur (Fig. 3a). We conclude that the pre-IC cannot assemble in the absence of RNAs, highlighting a defect in S-phase kinase-dependent activation of replication.

S-phase kinases phosphorylate several residues of the DNA helicase MCM2-7 complex, leading to its activation and to DNA unwinding at replication origins. Upon incubation with aphidicolin to inhibit DNA polymerases, the DNA helicase is uncoupled from DNA polymerases, and unwound DNA accumulates downstream of the replication fork, leading to RPA overloading on ssDNA^37,38^ (compare lanes 2 and 4, Fig. 3b). In RNase A-treated extracts, RPA loading was strongly impaired both in the presence or absence of aphidicolin (Fig. 3a and Fig. 3b, lanes 3 and 5), demonstrating that the MCM2-7 helicase could not be activated in the absence of RNAs. Moreover, the transfer of sperm nuclei assembled in a mock-treated egg extract to a RNA-depleted egg extract did not affect replication (Fig. 3c, lane B). This suggests that the helicase was already set for activation during the first step of incubation, and is consistent with the finding that DNA replication was not affected when the RNase treatment was delayed (Fig. 1a). Pre-IC assembly and DNA replication were restored when sperm chromatin assembled in RNase A-treated extracts was transferred to a mock-treated extract (Fig. 3c, lane C), but not to another RNase A-treated extract (Fig. 3c, lane D). Altogether, these data suggest that activation of the MCM2-7 helicase to allow the progression from pre-RC to pre-IC includes an RNase A-sensitive step.

To further confirm that the activation of S-phase kinases was RNA-dependent in the replication pathway, we investigated whether DNA replication required RNAs after CDC7 loading onto chromatin (Fig. 1c). To this aim, we supplemented egg extracts with p21^CIP1/Waf1^ that blocks DNA synthesis initiation by interacting with PCNA and inhibiting the activity of CDK2 and DNA polymerase delta, without affecting sperm chromatin decondensation and NE assembly^39–43^. Transfer of this sperm chromatin to a second mock-treated extract led to DNA replication resumption because CDK2 was no longer inhibited (Fig. 3d, lane A). Similarly, when chromatin from a p21-incubated extract was transferred to an RNase A-treated extract, DNA replication occurred because pre-RC were already formed (Fig. 3d, lane B). In this condition, CDC7 could bind to chromatin when incubated in the first extract. When this chromatin was transferred to an extract devoid of CDK inhibitor, RNase A had no effect on DNA replication, allowing CDK-dependent activation. Conversely, DNA replication did not occur in nuclei assembled in an RNase A-treated extract and then transferred to a p21-treated extract because pre-IC could not assemble. The RNase A-dependent inhibition of CDC7 recruitment to chromatin was reversible (Fig. 3d, lane C), thus confirming that CDC7 binding to chromatin can occur in the presence of p21^42^. An explanation of these results could be that nuclear import was affected in RNase-treated extracts. The level of chromatin-bound CDC7 (Fig. 3d, lane C) was indeed lower than in extracts with normal DNA replication, suggesting a delay in nuclear import following RNase A treatment. Thus, we investigated whether the NE was functional in the absence of RNAs.

### RNA depletion impairs nuclear import of replication factors

**Fig. 4:**
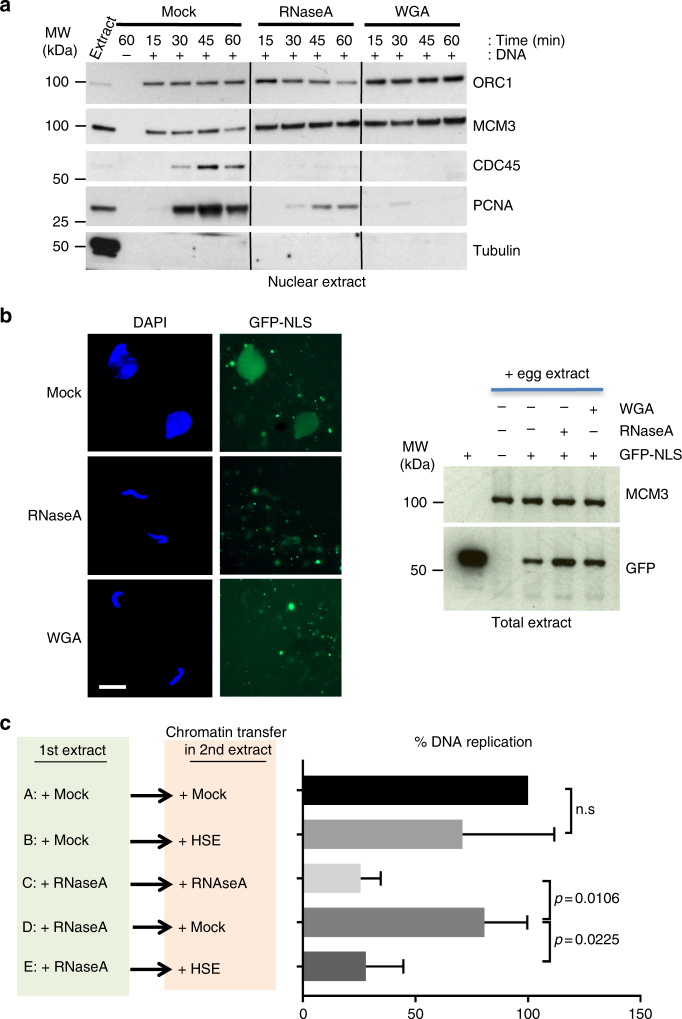
RNAs are required for nuclear import mediated by functional nuclear envelope assembly. **a** Sperm nuclei were incubated in mock- and RNA-depleted extracts. At the indicated time points, nuclei were purified and nuclear extracts analysed by western blotting. The anti-tubulin antibody was used to monitor cytoplasmic contamination during nuclear extract preparation. Addition to the extracts of wheat germ agglutinin (WGA), which inhibits nuclear import, blocks accumulation of factors associated with replication activation and elongation (CDC45 and PCNA, respectively). **b** Sperm nuclei were incubated in egg extracts supplemented with GFP-NLS (green) to monitor nuclear import. Its accumulation into nuclei (stained with DAPI) was monitored with an epifluorescence microscope. Scale bar, 15 μm. GFP-NLS level in each condition was assessed by western blotting analysis of total extracts. MCM3 was used as loading control. **c** Chromatin was assembled in mock- or RNase A-treated LSE (for 20 min) and then transferred to a mock- or RNAse A-treated LSE, or to a HSE, which lacks membranes. The graph shows the percentage of DNA replication at 120 min after transfer to the second extract (*n* = 3). Error bar shows the SD. *P*-values were determined by using a two-tailed Student’s *t*-test

An important role of the nuclear membrane is to ensure the import and concentration of factors involved in the activation of DNA replication, a step normally accompanied by nuclear swelling^13,44–46^. The small size of sperm nuclei in RNA-depleted egg extracts could be the consequence of nuclear swelling inhibition. Therefore, we investigated whether the observed DNA replication inhibition could be linked to impaired nuclear concentration of replication initiation factors, specifically those involved in DNA replication activation. Western blot analysis indicated that ORC1 and MCM3 levels were not affected in nuclei assembled in egg extracts incubated with RNase A or supplemented with wheat germ agglutinin (WGA), a well-known nuclear import inhibitor^47^ (Fig. 4a). This is consistent with the findings that pre-RC assembly can occur in the absence of nuclear membrane components^13,37,48^, from the end of mitosis. This is also in agreement with the finding that pre-RC formation was not inhibited in RNA-depleted extracts. Conversely, factors associated with DNA replication activation, such as CDC45 and PCNA, did not concentrate in nuclei formed in RNase A- or WGA-treated extracts (Fig. 4a). This suggested a nuclear import defect in RNase-treated extracts, and explained the defect in DNA replication activation.

To confirm this result, we assessed the nuclear import of GFP-NLS (Green Fluorescent Protein tagged with a Nuclear Localisation Signal motif) that was equally added to the extracts for each condition (Fig. 4b). GFP-NLS was not imported in nuclei assembled in RNase A- or WGA-treated egg extracts, confirming that RNAs are needed for proper nuclear organisation to allow the import and concentration of factors involved in DNA replication activation. Differently from what observed with wild-type RNase A, GFP-NLS accumulated normally in nuclei formed in extracts incubated with the catalytically inactive RNase A mutant (Supplementary Fig. 4b).

This result could explain the rescue of DNA replication when nuclei assembled in a RNA-depleted extract were transferred into a mock egg extract (Fig. 3c), because a functional NE could form in the second extract. In agreement, transfer of nuclei pre-assembled in a RNA-depleted extract into a HSE, which lacks nuclear membrane vesicles, did not restore DNA replication (Fig. 4c, lane E). Conversely, transfer in a mock-treated extract, which contains the nuclear membrane fraction, rescued DNA replication (Fig. 4c, lane D).

Together, these results show that RNAs are required for the formation of a functional NE that can concentrate nuclear factors involved in DNA replication activation.

### RNAs mediate NPC assembly in an ELYS-dependent manner

**Fig. 5:**
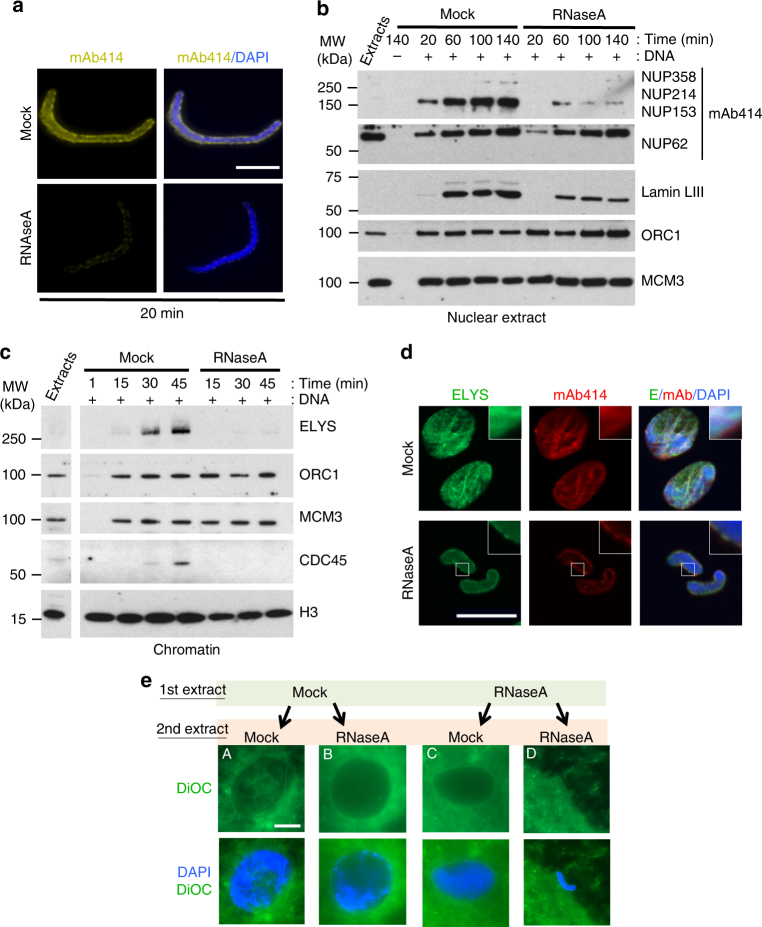
RNAs are required for nuclear pore complex assembly and nuclear import. **a** Representative images of immunofluorescence analysis of nuclei fixed after incubation in mock- or RNase A-treated *X*. *laevis* egg extracts for 20 min. The mouse monoclonal antibody 414 (mAb414) recognises several nucleoporins (NUP358, 214, 153, 62); DNA was stained with DAPI (blue). Scale bar, 10 μm. **b** Western blot analysis of nuclear extracts after incubation in mock- or RNA-depleted egg extracts for the indicated time. **c** Sperm nuclei were incubated in mock- or RNA-depleted egg extracts. At the indicated time points, chromatin was isolated and immunoblotted with the indicated antibodies. **d** Immunofluorescence analysis of ELYS (green) and NUPs (red) expression in a representative population of nuclei assembled in mock- or RNase A-treated extracts for 60 min. Images were acquired with an ApoTome microscope. Scale bar, 15 μm. **e** Nuclear swelling and DNA decompaction are restored when nuclei are first incubated in RNase A-treated extracts and then transferred to mock-treated extracts (as explained in Fig. 3c). After transfer into the second extract, nuclei were fixed and stained with DAPI and DiOC_6_ to monitor decompaction and nuclear swelling. Scale bar, 10 µm

Nuclear import is mainly governed by a subset of proteins called NUPs that form NPCs. Analysis of NPC assembly by immunofluorescence staining and western blotting using a mouse monoclonal antibody (mAb414) against several NUPs (NUPs 358, 214, 153 and 62) showed that 20 min after sperm chromatin addition, NUPs could be detected on the rim of nuclei in mock-treated, but not in RNase A-treated egg extracts (Fig. 5a). Western blot analysis showed that NUP153, which localises at the pore nuclear interface, did not accumulate in nuclei from RNA-depleted extracts (Fig. 5b). Conversely, the level of NUP62, a component of the central NPC structure^49^, was only partially affected compared with ORC1 level. NUP214 and NUP358, which are located on the NPC cytoplasmic face, were barely detectable in both experimental conditions. This result showed that correct NPC assembly is RNA-mediated, and explains why the nuclear membrane cannot efficiently support nuclear import in nuclei formed in RNA-depleted egg extracts.

In vertebrates, ELYS/MEL-28 is a major chromatin component that coordinates DNA replication and nuclear assembly during nuclei reorganisation^9–11^. It is the earliest factor in the mitotic NPC assembly pathway that binds to decondensing chromatin and anchors NPC components^9,11,50^. Intriguingly, when ELYS recruitment to chromatin is impaired, smaller nuclei are formed^51^, like in RNA-depleted egg extracts. Therefore, we hypothesised that RNA depletion could inhibit ELYS binding to chromatin. Analysis of the proteins bound to chromatin assembled in RNA-depleted extracts confirmed that ELYS recruitment was strongly impaired compared with mock samples (Fig. 5c). Immunofluorescence analysis showed that the remaining ELYS was still at the nuclear rim and diffuse compared with its specific localisation in control nuclei (Fig. 5d), as previously shown^10^.

Moreover, the transfer of nuclei assembled in an RNA-depleted extract into a mock-treated extract not only restored DNA replication (Figs. 3c and 4c), but also rescued nuclear swelling, compared with transfer into another RNase A-treated extract (Fig. 5e). Together, these results confirm that in *X*. *laevis* egg extracts, RNAs are needed for the complete assembly of NPC to the nuclear rim.

### Chromatin accessibility and compaction are RNA-dependant

**Fig. 6:**
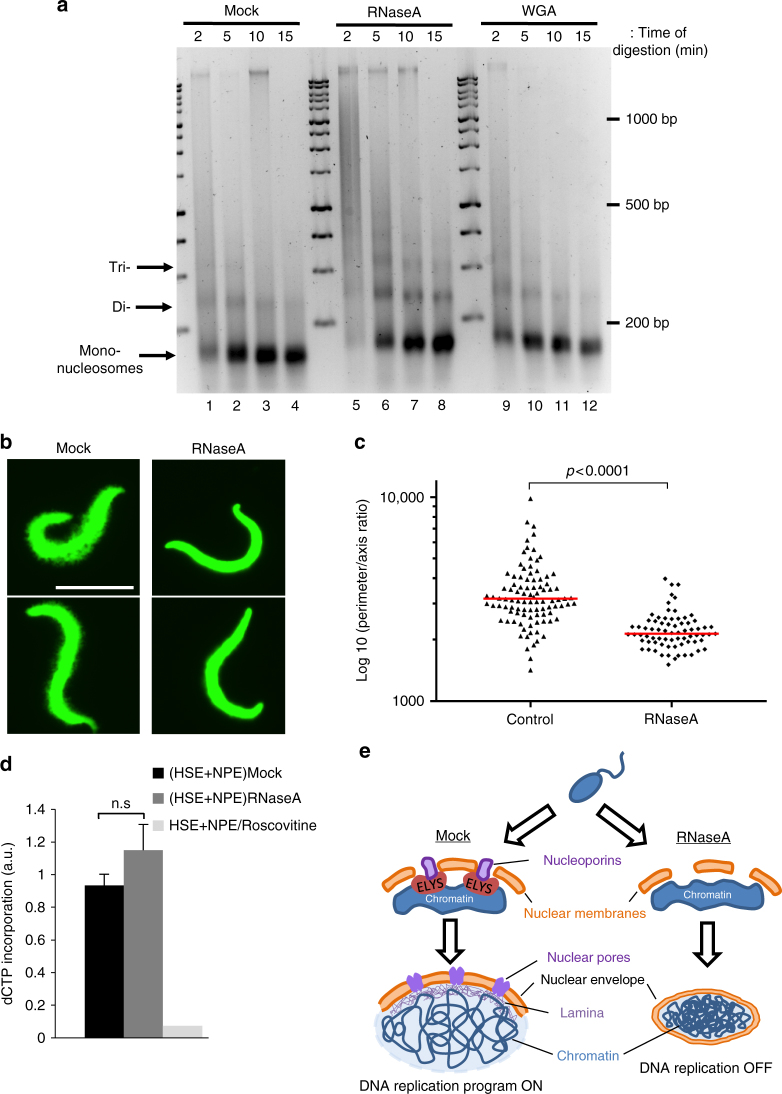
RNAs modulate the higher-order chromatin structure. **a** Micrococcal nuclease (MNase) digestion assays of nuclei that were assembled in mock- and RNA-depleted extracts for 15 min. Black arrows indicate the ladder of mono-, di- and tri-nucleosomes. **b** Immunofluorescence images of histone H2B in nuclei treated with a moderate salt concentration after incubation in mock- or RNA-depleted *X*. *laevis* egg extracts for 15 min. Scale bar, 17 μm. **c** Quantification of the chromatin perimeter-to-axis ratio in one representative experiment out of three. Mock-depleted extracts: *n* = 102 nuclei; RNase A-depleted extracts: *n* = 93 nuclei. A two-tailed Student *t*-test was performed to determine the *p*-value. **d** Quantification of three independent DNA replication assays (mean ± SD) in which sperm nuclei were incubated in mock or RNase A-treated HSE (for replication licensing) and then transferred to mock- or RNase A-treated NPE (for replication initiation). Nuclear membrane-free replication was monitored by autoradiography. A two-tailed Student’s *t*-test was performed to calculate the *p*-values; n.s., not significant. **e** Stepwise model of the major events leading to the replication initiation defect observed upon RNA depletion in egg extracts. Protamine removal and histone incorporation, two major steps during chromatin remodelling following fertilisation, occur normally both in the presence or absence of maternal RNAs. Newly formed chromatin in RNA-depleted extracts acquires a compact architectural organisation compared with control (mock-depleted) extracts. While licensing (pre-RC assembly) occurs normally, ELYS binding to condensed chromatin of nuclei assembled in RNA-depleted extracts is repressed. As a consequence, its accumulation at the nuclear rim is disturbed, and NPC assembly is altered. Nuclear pores are not incorporated in the NE membranes. Nuclear import is impaired and the accumulation of factors required for DNA replication activation is inhibited

Recent studies showed that proper nucleosome assembly is needed for ELYS recruitment to DNA^15,16^. Therefore, we asked whether the defects observed in RNase-treated extracts affected chromatin organisation. We monitored nucleosome assembly by micrococcal nuclease (MNase) digestion of sperm chromatin after incubation in *X*. *laevis* egg extracts for 15 min (before NE formation). In control samples, MNase digestion resulted in a multi- to mono-nucleosome ladder pattern (Fig. 6a, lanes 1–4). In chromatin assembled in RNase A-treated extracts, MNase digestion led to a similar nucleosome ladder pattern (Fig. 6a, lanes 5–8), but digestion was always less efficient, as shown by the presence of di- and tri-nucleosomes (lane 6–8), compared with control chromatin (lanes 2–4). This lower accessibility to MNase digestion was in agreement with the smaller size (Fig. 1d) and higher level of chromatin compaction observed by electron microscopy in nuclei assembled in RNA-depleted extracts compared with controls (Supplementary Fig. 5). Noticeably, MNase accessibility to chromatin assembled in WGA-treated extracts was similar to what observed in control (lane 9–12), ruling out the possibility that a nuclear import defect affects chromatin compaction. Our results are also in agreement with the finding that in *Drosophila* S2 cells, pre-incubation with RNase reduces chromatin sensitivity to MNase digestion^20,52^.

To further investigate chromatin compaction in RNase-treated extracts, we isolated nuclei at a moderate salt concentration (0.2 M KCl) to preserve core histones (see ref.^53^). Immunofluorescence analysis of histone H2B (Fig. 6b) showed that sperm chromatin from mock-treated nuclei was relaxed and chromatin microvilli could be detected in the nucleus core region. In chromatin from RNase A-treated extracts, this relaxation step was strongly reduced. We tried to quantify this phenotype, and calculated the ratio between the chromatin perimeter and its axis (in yellow and red, respectively, Supplementary Fig. 5e). The results confirmed that chromatin from RNA-depleted extracts was more compact than in control (Fig. 6c). Together with the MNase digestion profiles, these results confirm that in *X. laevis* egg extracts, RNAs are needed for efficient chromatin decompaction.

If impaired ELYS recruitment to chromatin were the main mechanism by which RNA depletion affected DNA replication, the use of concentrated membrane-free nucleoplasmic egg extracts (NPE), in which sperm DNA can replicate in a nucleus-free environment^13^, should overcome the replication defect. In this assay, we first incubated sperm nuclei in mock-treated HSE that support pre-RC formation but not nuclear assembly, and then we added the NPE^13^. In this condition, DNA replication occurred because the NPE mimicked the local concentration of replication factors needed to promote replication initiation^13^ (Fig. 6d). In agreement, addition of the CDK inhibitor roscovitine to NPE blocked DNA replication initiation. DNA replication also occurred normally when RNase A-treated HSE and NPE were used. These results showed that in conditions where replication can occur without nuclear membrane, RNase A has no effect. On the basis of all these data, we concluded that RNAs are involved in the coordination of chromatin accessibility and DNA replication activation through ELYS (Fig. 6e).

## Discussion

In this study, we investigated the role of RNAs during the transient steps that establish the ability of packed sperm DNA to acquire a nuclear structure and prepare the genome for duplication following fertilisation. Our results were obtained using *X. laevis* egg extracts that are transcriptionally quiescent and in which translation is dispensable for DNA synthesis. They show that RNA components are required to promote ELYS-dependent DNA replication activation. It was previously reported that ELYS binding to chromatin initiates NPC formation independently of NE assembly^9,10,51^ and it was concluded that ELYS coordinates NPC assembly with DNA replication regulation. Consistent with this idea, ELYS immunodepletion from *X. laevis* egg extracts leads to the formation of small nuclei that do not swell and cannot replicate their genome^9,10^. Here, we observed a similar phenotype in nuclei assembled in RNA-depleted *X*. *laevis* egg extracts. Our data are consistent with a model in which the absence of RNAs impairs ELYS binding to chromatin and blocks DNA replication activation. This is not caused by a defect in pre-RC assembly, but in pre-RC activation that is the consequence of the failure to import and sufficiently concentrate S-phase kinases, such as CDC7, in the nucleus. Interestingly, nuclei formed in CDK2-depleted *X*. *laevis* egg extracts also are smaller than control nuclei^43^. In agreement, RNA depletion does not affect DNA replication when the need for a functional nuclear membrane assembly is bypassed (experiments with NPE). Similarly, once the nucleus is formed, DNA replication is not affected by RNA depletion.

We attempted to rescue DNA replication by re-introducing purified RNAs in RNA-depleted egg extracts. However, the results of these experiments were inconclusive, most likely because the functional/structural integrity of the re-introduced purified RNAs was compromised. As an alternative, we used chromatin transfer experiments to demonstrate that the phenotype is reversible. As protein synthesis was blocked by addition of cycloheximide in the egg extracts, our experiments indicate that maternal RNAs with non-coding functions are important for NE assembly.

Previous work showed that ELYS binding to chromatin and its accumulation at the nuclear rim are possible only if nucleosomes are correctly assembled. Indeed, ELYS interacts directly with histone H2A-H2B and, consequently, nucleosome loss in *X*. *laevis* egg extracts or in mouse zygotes prevents NPC assembly^15,16^. Our data show that RNA depletion does not hinder protamine removal and maternal histone incorporation in nucleosomes. Thus, nucleosome alterations cannot explain ELYS binding defect in our experimental conditions. As H2A-H2B and ELYS can interact directly in vitro^16^, it is unlikely that a subset of RNAs is required to target ELYS directly to chromatin. However, our data show that chromatin remains more compact when it is assembled in RNA-depleted egg extracts. Two distinct consecutive steps of sperm chromatin decondensation have been characterised following fertilisation^8^. The earliest, which occurs immediately after addition of sperm nuclei to egg extracts, ensures the nucleoplasmin- and nucleophosmin-mediated exchanges of sperm protamines with maternal histones, ultimately resulting in chromatin relaxation. The second step occurs after NE assembly and depends on nuclear import. As interfering with ELYS binding to chromatin generates small nuclei with compact chromatin^9,10,51^, this effect could be related to a defect in the second step of decondensation owing to inefficient nuclear import. In our experiments, higher chromatin compaction, as indicated by resistance to MNase digestion, was observed already after 15 min of incubation in RNase A-treated *X*. *laevis* egg extracts. At this time, NPCs were not assembled and nuclear import was not initiated. In addition, nuclear import inhibition using WGA could not account for the chromatin resistance to MNase digestion. Therefore, we believe that RNAs are required after the chromatin-remodelling step to complete decondensation of sperm chromatin during fertilisation, resulting in a fully decompacted genome. We show that this step, which contributes to the acquisition of a nuclear structure competent for DNA replication, is mediated by ELYS, but is independent of pre-RC assembly. In agreement with these results, independent studies have demonstrated that a subset of chromatin-associated RNAs is needed to preserve the open chromatin state of euchromatic regions in mammalian somatic cells and *Drosophila* late-stage embryos^19,52,54^. Our data are also in line with the recent finding that loss of the lysine demethylase LSD1 activity alters the re-establishment of the proper organisation of interphasic chromatin at the end of mitosis in *X*. *laevis* egg extracts^55^. Chromatin assembled in such conditions can form a nuclear envelope that is, however, not functional because NPC proteins, such as ELYS, are not recruited.

## Methods

### *X*. *laevis* egg extracts and DNA replication kinetics

*X. laevis* frogs were imported from the Centre de Ressources Biologiques Xenopes, University of Rennes, France. Experimental procedures and animal care and use were approved by the 'Direction Départementale des Services Vétérinaires'.

LSE were prepared as previously described^56^. Eggs were collected in HSB buffer 1X (15 mM HEPES pH 7.6, 110 mM NaCl, 2 mM KCl, 1 mM MgSO_4_, 0.5 mM Na_2_HPO_4_, 2 mM NaHCO_3_). Eggs were then dejellied in HSB buffer 0.3X, pH 7.9, containing 2% cysteine (w:v), and were washed twice in 1X MMR buffer (5 mM HEPES pH 7.8, 0.1 M NaCl, 2 mM KCl, 0.1 mM EDTA, 1 mM MgCl_2_, 2 mM CaCl_2_) before activation in 0.2X MMR supplemented with 0.3 μg ml^–1^  calcium ionophore. Packed eggs were crushed in XB buffer (detailed below) containing cytochalasin B (100 μg ml^–1^) by centrifugation (Sorvall HB6 swinging rotor) for 20 min at 10,000 rpm.  Low speed supernatants were finally clarified by centrifugation for 20 min at 20,000 rpm in an SW 55Ti rotor. Extracts were aliquoted and stored at  –80 °C. HSE and NucleoPlasmic Extracts (NPE) were a kind gift from H.M. Mahbubani (Cancer Research UK, Clare Hall Laboratory). Chromosomal DNA replication was assayed by adding demembranated *X*. *laevis* sperm nuclei to RNase A- or mock-treated egg extracts supplemented with [α–^32^P]-dCTP. Unless otherwise stated, mock-treated samples were incubated with ultrapure water. DNA synthesis was monitored by TCA precipitation. In brief, incorporated acid-insoluble material was spotted onto Whatman glass microfiber filters, grade GF/C, and then precipitated with 5% TCA solution containing 2% pyrophosphate. After ethanol washes, filters were dried and the incorporated TCA-precipitated radioactivity was counted in scintillation liquid. Alternatively, incorporation of radiolabelled deoxynucleotides in DNA was monitored by autoradiography following agarose gel electrophoresis of purified DNA. For the chromatin transfer experiments, replication kinetic values were normalised to the DNA replication value (set at 100%) in the mock sample. M13 replication kinetics was assessed using 400 ng of ssDNA per 50 µl of HSE^25^.

### Purification of chromatin and nuclei

Sperm chromatin purification after incubation in egg extracts was performed as previously described^56,57^. In brief, samples were diluted fourfold in XB (10 mM Hepes-KOH pH 7.7, 100 mM KCl, 0.1 mM CaCl_2_, 1 mM MgCl_2_, 50 mM sucrose) with 0.2% Triton X-100, incubated on ice for 5 min and centrifuged through a sucrose cushion. For the chromatin transfer experiments, chromatin samples were incubated in the first extract for 30 min. Purification for chromatin transfers and isolation of nuclei were performed by mixing samples with five volumes of CPB (20 mM Hepes-KOH pH 7.7, 50 mM KCl, 5 mM MgCl_2_, 2% sucrose) instead of XB/Triton X-100. Chromatin pellets were resuspended in 2× LB (0.125 M Tris-HCl pH 6.8, 4% SDS, 20% glycerol, 10% 2-β-mercaptoethanol and 0.004% bromophenol blue), denatured at 95 °C for 5 min and then stored at −20 °C or immediately analysed by SDS-PAGE, using gradient Bis-Tris gels (Thermo Fisher Scientific). Histone extraction was performed by acid extraction of chromatin using 0.4 N H_2_SO_4_. The soluble fraction containing histones was then TCA-precipitated and the pellet washed with ice-cold acetone. Histone pellets were dissolved in H_2_O and analysed on SDS-PAGE gels.

### Fluorescence and immunofluorescence microscopy analysis

Sperm nuclei incubated in *X*. *laevis* egg extracts were supplemented with Cy3-dCTP and 2 μl of this solution was mounted between slide and coverslip in fixing solution (45 mM PIPES pH 7.2, 45 mM NaCl, 240 mM KCl, 10% formalin, 50% glycerol, 2 μg ml^−1^ Hoechst and 3,3′-dihexyloxacarbocyanine (DiOC_6_)) to visualise DNA and vesicle membrane fusion. Slides were analysed by fluorescence microscopy. Immunofluorescence staining was performed as follows. The reaction mixture was diluted 10 times in XB buffer, and nuclei were spun on coverslips through a 0.5 M sucrose cushion at 100×*g* for 10 min. Nuclei on coverslips were fixed in 4% paraformaldehyde for 10 min and then washed with PBS/0.1% Tween 20 before blocking with 3% BSA in PBS/0.1% Tween 20. Incubation with primary antibodies was performed at 4 °C and overnight, while incubation with secondary antibodies was performed at room temperature (RT) for 1 h. ProLong Gold (Thermo Fisher Scientific) was used for slide mounting. A Zeiss Axioimager ApoTome microscope was used for image acquisition and ImageJ for image analysis.

### Antibodies

The antibodies used in this work were against: H3 (Abcam, ab1791, dilution 1/2000), H2B (Abcam, ab1790, dilution 1/2000), NUPs (Abcam, mab414, dilution 1/100), SMC1 (Bethyl, A300-055A, dilution 1/1000), phosphorylated CHK1 (Cell Signaling, 2341 S), PCNA (Sigma, P8825, dilution 1/2500), DNA polymerase alpha (Abcam, ab31777, dilution 1/500), RPA32^37^ (dilution 1/500) Geminin, CDT1, ORC2^56^ (dilution 1/1000), MCM3^57^ (dilution 1/2000), CDC45^58^ (dilution 1/1000), ELYS^9,10^ (dilution 1/50) and CDC7^59^ (dilution 1/1000).

### Micrococcal nuclease digestion

Sperm nuclear assembly was stopped by diluting the reaction mixture with ice-cold XN buffer (50 mM Hepes-KOH pH 7.0, 250 mM sucrose, 75 mM NaCl, 0.5 mM spermidine and 0.15 mM spermine) and then nuclei were pelleted by centrifugation at 3000×*g* for 5 min. Nuclei were resuspended in MNase buffer (10 mM Tris-HCl pH 7.5, 80 mM NaCl, 25% glycerol) containing 50 units ml^−1^ of micrococcal nuclease and then incubated at RT. At different time points, samples were taken and the enzymatic reaction stopped by addition of 30 mM EDTA. DNA was then extracted and run on a 1.8% agarose gel in 0.5× TBE. The nucleosome ladder was detected using the SYBR Gold nucleic acid gel stain (Thermo Fisher Scientific).

### Electron microscopy

Sperm nuclei were immersed in a solution of 2.5% glutaraldehyde in 1× PHEM (60 mM PIPES, 25 mM HEPES, 10 mM EGTA, 4 mM MgSO_4_) buffer (pH 7.4) at 4 °C. Then, they were rinsed in PHEM buffer and post-fixed in 0.5% osmic acid in the dark at RT for 2 h. After two rinses in PHEM buffer, cells were dehydrated in a graded series of ethanol solutions (30–100%) and embedded in EmBed 812 using an Automated Microwave Tissue Processor for Electronic Microscopy, Leica EM AMW. Thin sections (70 nm; Leica-Reichert Ultracut E) were cut at different levels of each block. Sections were counterstained with 1.5% uranyl acetate in 70% ethanol and lead citrate and observed using a Tecnai F20 transmission electron microscope at 200KV at the CoMET MRI facility, INM, France.

### Recombinant proteins

Plasmids encoding wild-type pancreatic bovine RNase A and the inactive H12K/H119Q mutant were a gift from E. Boix **(**Universitat Autònoma de Barcelona). Proteins were expressed in *E*. *coli* BL21 (AI) cells after induction with 1 mM IPTG and 0.2% arabinose at 37 °C for 4 h. The protocol to purify the recombinant proteins recovered from inclusion bodies was described previously^60^. We resuspended inclusion bodies in a denaturing buffer containing 50 mM Tris HCl pH 8, 5 M guanidine hydrochloride, 5 mM EDTA and protease inhibitors cocktail. The mixture was sonicated, mixed during 1 hour at 4 °C and finally centrifuged for 30 min at 12,000×*g*. Soluble proteins were resuspended in 5 mM Tris HCl pH 8.0, 1 mM DTT, 20% glycerol. Purified proteins were further concentrated and incubated for 10 min at 95 °C and slowly renatured at room temperature. The supernatants containing the recombinant RNAses were recovered following a 30 min centrifugation at 13,000 rpm. Recombinant wild-type and mutant RNase A were purified in parallel and dialysed against 5 mM Tris pH 7.0 before addition to the egg extracts at the final concentration of 250 ng μl^−1^.

### Data availability

Data supporting the findings of this study are available within the paper and its supplementary information files, including uncropped scans of the most important blots. All data are available from the authors upon reasonable request.

## Electronic supplementary material

Supplementary Information
