# Author Correction: RNAs coordinate nuclear envelope assembly and DNA replication through ELYS recruitment to chromatin

**DOI:** 10.1038/s41467-018-02968-9

**Published:** 2018-02-05

**Authors:** Antoine Aze, Michalis Fragkos, Stéphane Bocquet, Julien Cau, Marcel Méchali

**Affiliations:** 1 Institute of Human Genetics, UMR 9002, CNRS and the University of Montpellier, Replication and Genome Dynamics, 141 rue de la Cardonille, 34396 Montpellier, France; 2 Institute of Human Genetics, UMR 9002, CNRS and the University of Montpellier, Montpellier RIO Imaging, 141 rue de la Cardonille, 34396 Montpellier, France; 3 Present Address: Institut Gustave Roussy, Genetic Stability and Oncogenesis Department, 39 rue Camille Desmoulins, 94805 Villejuif, France

Correction to: *Nature Communications* (2017) 10.1038/s41467-017-02180-1, Article published online 14 December 2017

In the original version of this Article, the affiliation details for Antoine Aze, Michalis Fragkos, Stéphane Bocquet, Julien Cau and Marcel Méchali incorrectly omitted ‘CNRS and the University of Montpellier’. This has now been corrected in both the PDF and HTML versions of the Article.

