# Supplementary material for: RNAs coordinate nuclear envelope assembly and DNA replication through ELYS recruitment to chromatin
Source: Nat Commun. 2017 Dec 14;8:2130. doi: 10.1038/s41467-017-02180-1 (PMC5730577; doi:10.1038/s41467-017-02180-1)
Supplement: Supplementary file 1 — Supplementary Information [file 41467_2017_2180_MOESM1_ESM.pdf]

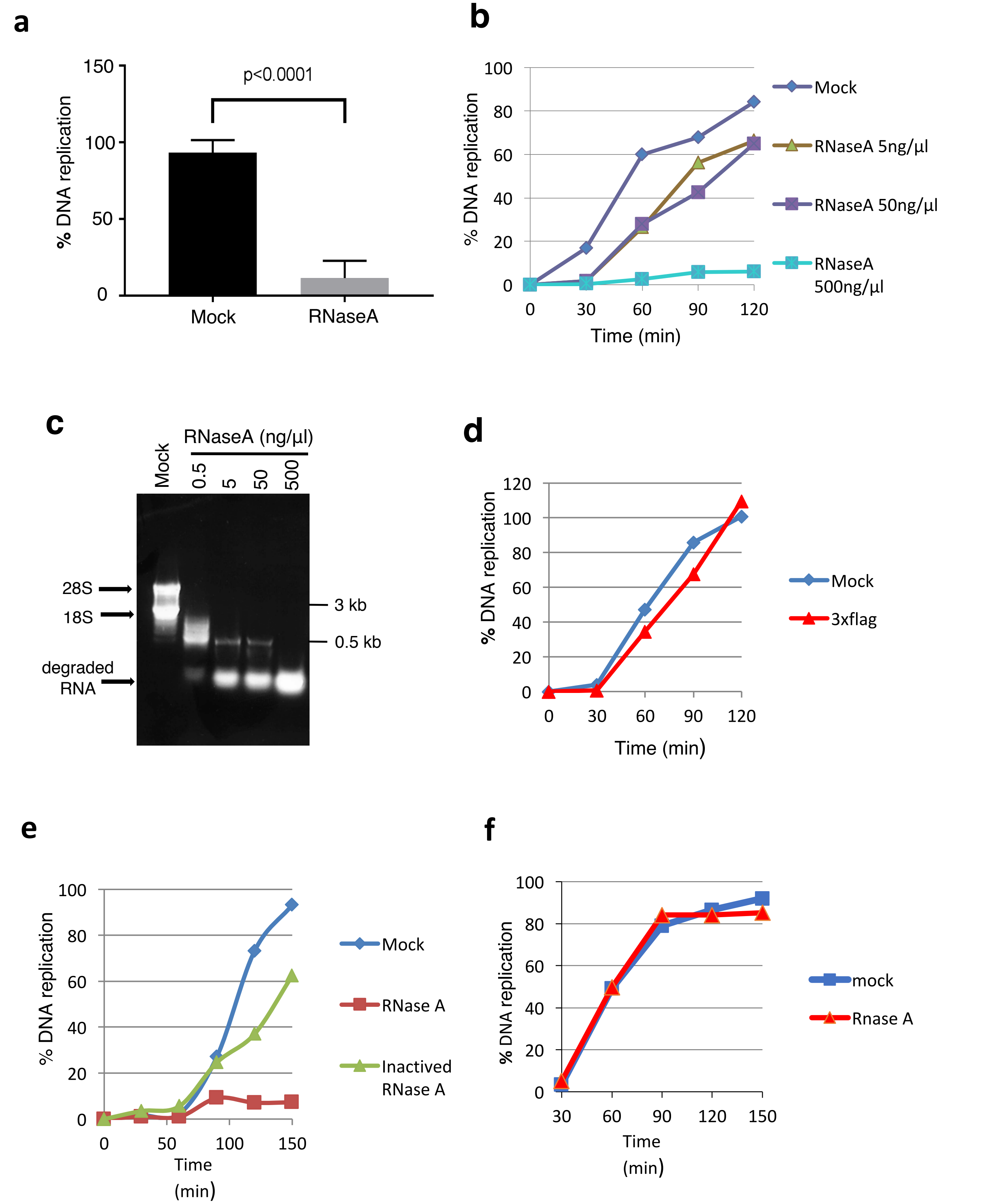

Supplementary Figure 1: Dose-response effect of RNase A treatment on DNA replication.

(**a**) Average DNA replication efficiency (mean + SD) of nuclei incubated in RNase A-treated extracts when all nuclei are replicated in mock treated extracts (n=6). P-values were calculated using the two-tailed Student's *t*-test. (**b**) Replication kinetics of sperm nuclei in LSE that were pre-incubated with the indicated amounts of RNase A. Mock: LSE incubated with the same volume of ultrapure H_2O. (**c**) Agarose gel (2%) showing the efficiency of RNA degradation in egg extracts after incubation with RNase A. (**d**) Incubation with a similar amount (500 ng/µl) of 3x-FLAG peptide has no effect on DNA replication. (**e**) Heat-inactivated RNase A does not inhibit DNA replication. RNase A was heat-inactivated at 95°C for 60min and cooled on ice before addition to the egg extracts. (**f**) RNase treatment of sperm nuclei before incubation in the extract does not affect DNA replication. Sperm nuclei were pre-incubated with RNase A for 20min, then washed and transferred into fresh egg extracts for DNA replication monitoring.

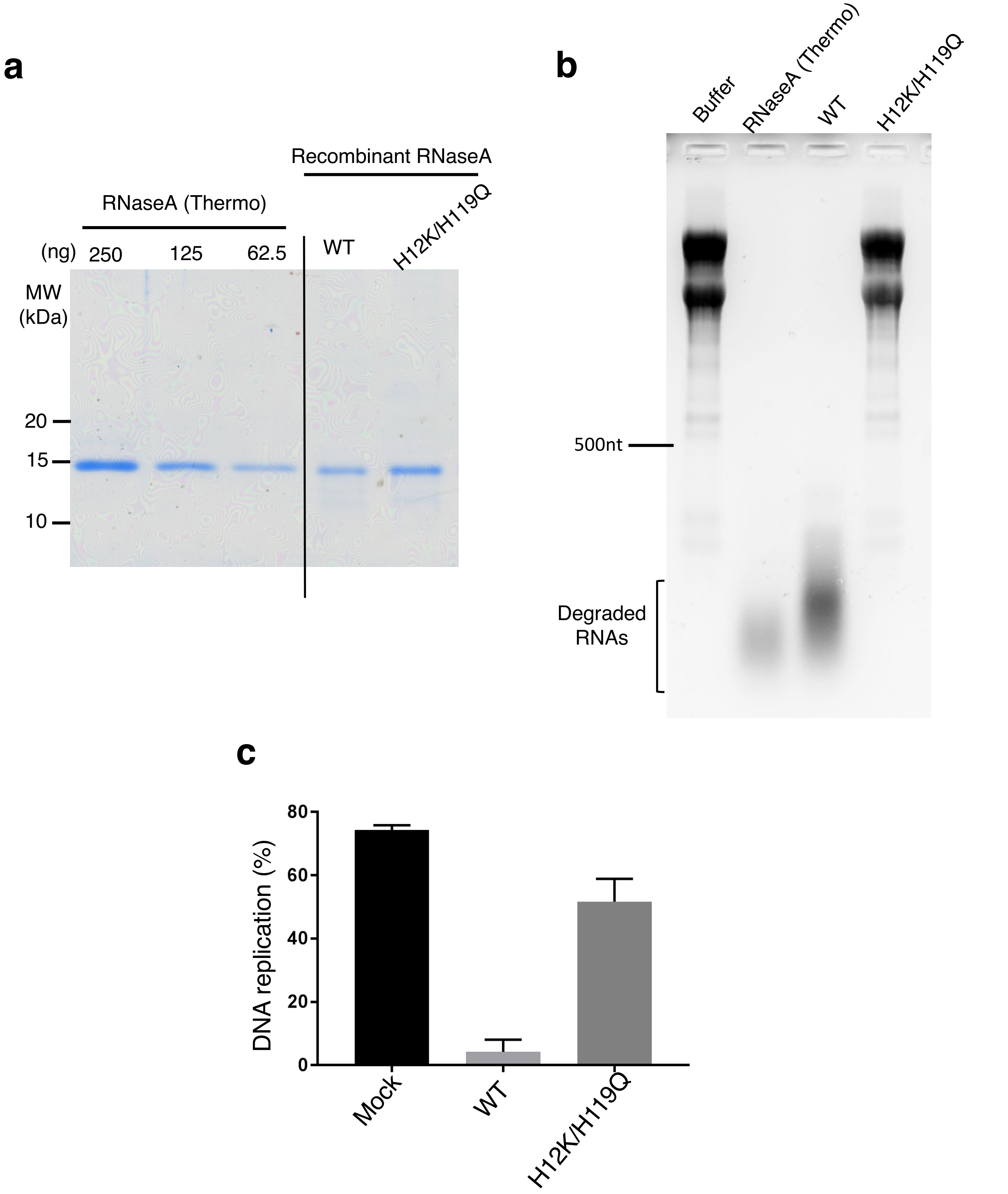

Supplementary Figure 2: Nuclear processes are blocked by RNase A activity.
(**a**) Recombinant wild type RNase A (WT) and the catalytically inactive H12K/H119Q mutant were purified from bacteria and then analysed by SDS-PAGE followed by Coomassie blue staining. Different amount of RNase A from Thermofisher were used for comparison. (**b**) Assessment of the ability of purified recombinant RNase A to degrade RNAs in *X. laevis* egg extracts. Egg extracts were incubated with the RNase A used in the study (Thermo), recombinant WT RNase A or the H12K/H119Q mutant at 23°C for 15min. RNAs were then isolated, separated on denaturing agarose gel and detected by staining with GelRed™. (**c**) The catalytically inactive RNase A mutant does not impair DNA replication. Sperm nuclei were incubated in extracts pre-incubated with recombinant WT RNase A or the H12K/H119Q inactive mutant and DNA replication was monitored. The shown data represent the mean replication efficiency from 3 independent experiments (error bars= SD).

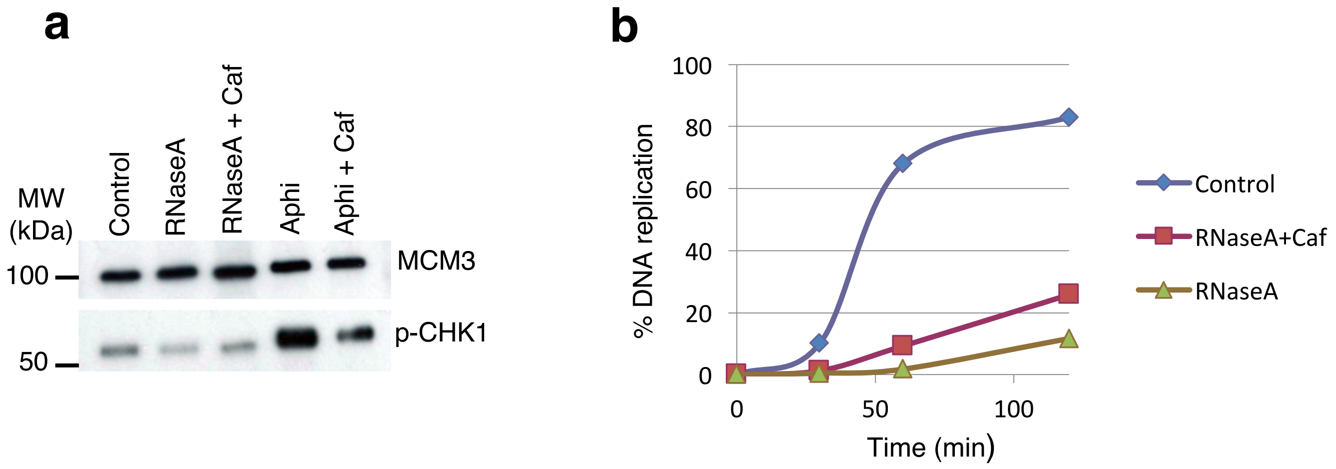

Supplementary Figure 3: RNase A does not activate the checkpoint response.
(**a**) Western blot analysis of nucleoplasmic fractions of sperm nuclei formed in RNase A-treated LSE, in the presence or absence of caffeine (Caf). Aphidicolin (Aphi) was used to induce the DNA damage response. Detection of phosphorylated CHK1 was used to test checkpoint activation. Control sample was incubated with caffeine. MCM3 was used as loading control. (**b**) Caffeine does not alleviate the inhibitory effect of RNase A on replication. Replication was monitored in 250 ng/ µl RNase A-treated LSEs in the presence or absence of 5mM caffeine.

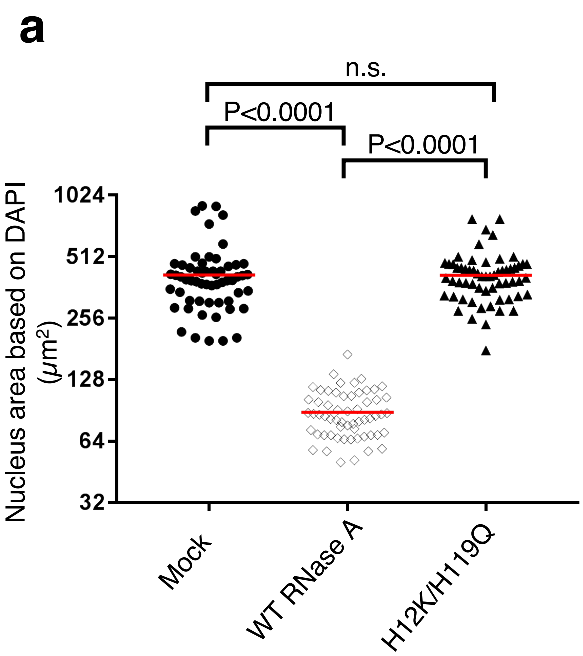

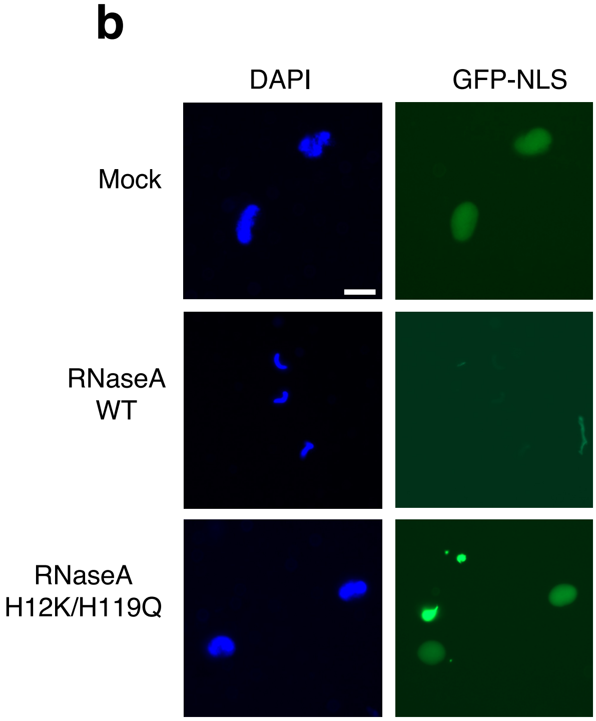

Supplementary Figure 4: Nuclear growth and nuclear import are not affected by incubation with a catalytically inactive RNase A.
(**a**) The catalytically inactive RNase A mutant does not affect nuclear growth. Nuclei were assembled in extracts pre-treated with recombinant WT RNase A or the H12K/H119Q inactive mutant. The nuclear size of about 125 nuclei per condition was then measured based on the DAPI staining area. Red bar shows the mean value. A two-tailed Student's *t*-test was performed to determine the p-values. (**b**) GFP-NLS was added to extracts after incubation with mock (buffer), wild type (WT) recombinant RNase A or the H12K/H119Q mutant. GFP-NLS nuclear accumulation was then monitored by epifluorescence microscopy. Scale bar, 27 µm

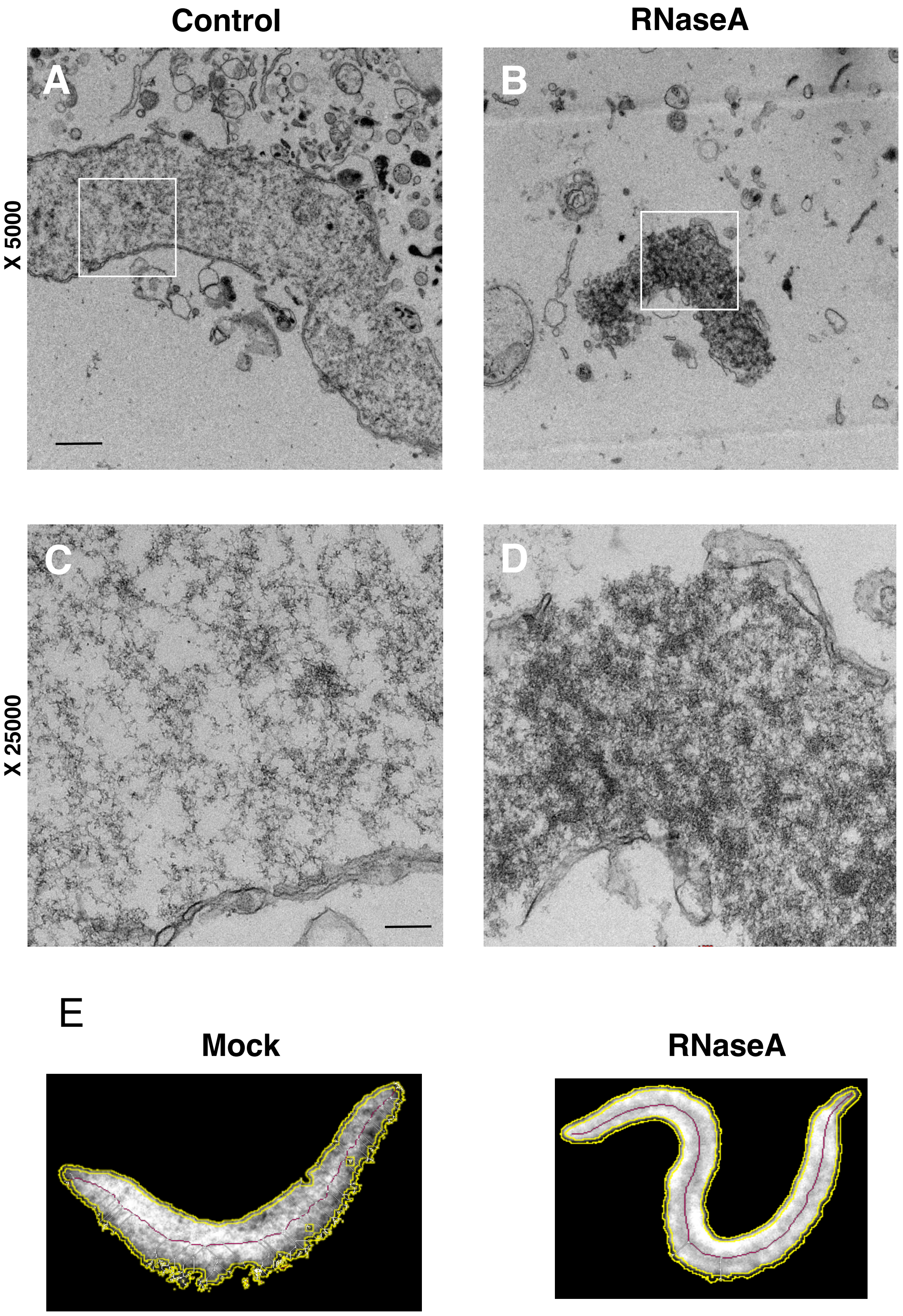

Supplementary Figure 5: RNA depletion leads to a defect in chromatin compaction.

A-B: Electron microscopy images of thin sections of sperm heads fixed after incubation in mock- or RNase A-treated *X. laevis* egg extracts for 15min (A and B respectively). Scale bar: 1 µm. Magnification: x5000.

C-D: Enlargement of the areas delimited by the white frame in A and B, respectively. Differently from C, chromatin density in D shows a high level of compaction. Scale bar: 200 nm. Magnification: x25000.

E: Measurement of microvilli from sperm nuclei by quantifying the chromatin perimeter. Two parameters (perimeter and main axis, in yellow and red respectively) were measured for the morphological analysis of nuclei assembled in mock- or RNase A-treated extracts after extraction with moderate salt concentration (see Fig. 5b). The perimeter-to-axis ratio was used for the graph in Fig 5c. Chromatin was detected by immunofluorescence staining using an anti-H2B antibody.

For Figure 5C

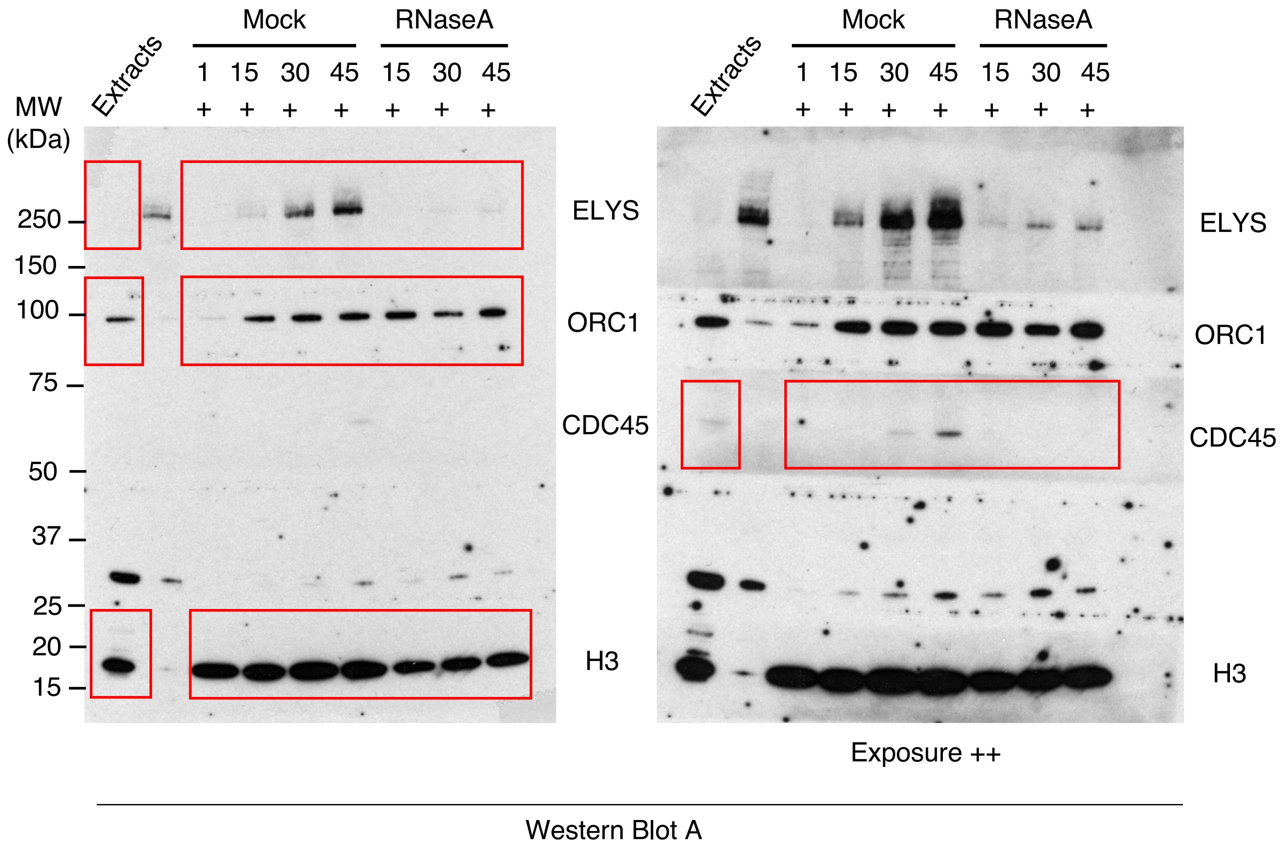

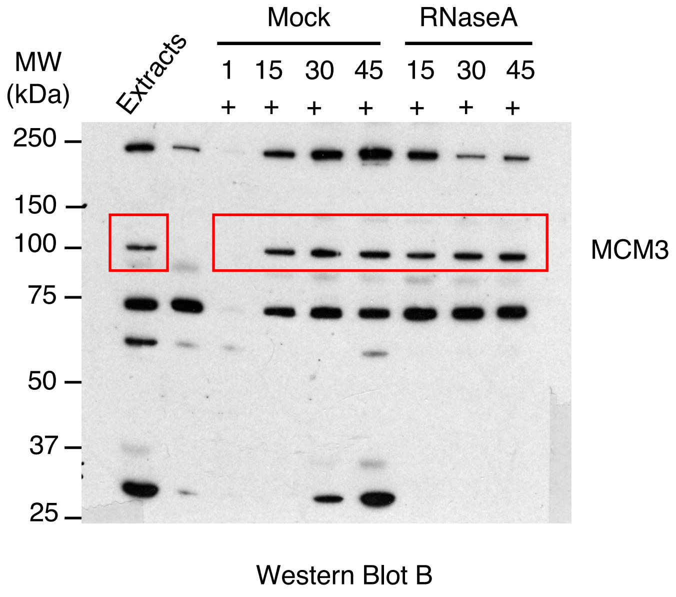

Supplementary Figure 6: Uncropped blots

SDS polyacrylamide gels for Westerns Blot A and B were loaded with the same samples from the same experiment.
Boxed areas correspond to images presented in the indicated main text.
